# Molecular abnormalities in autopsied brain tissue from the inferior horn of the lateral ventricles of nonagenarians and Alzheimer disease patients

**DOI:** 10.1186/s12883-020-01849-3

**Published:** 2020-08-27

**Authors:** Andrew Pearson, Rosa Ajoy, Gogce Crynen, Jon M. Reed, Moustafa Algamal, Michael Mullan, Dushyant Purohit, Fiona Crawford, Joseph O. Ojo

**Affiliations:** 1 Roskamp Institute, Sarasota, Florida 34243 USA; 2 The Open University, Milton Keynes, UK; 3 Boehringer Ingelheim Pharmaceuticals, Inc., Ridgefield, CT 06877 USA; 4 Bronx Veteran Administration Hospital, Bronx, NY 10468 USA; 5 Neuropathology Division, Department of Pathology, Mount Sinai School of Medicine, New York, NY 10029 USA

**Keywords:** Choroid plexus, Ependymal cells, Lateral ventricles, Alzheimer’s disease, Nonagenarians, Tau, Amyloid

## Abstract

**Background:**

The ventricular system plays a vital role in blood-cerebrospinal fluid (CSF) exchange and interstitial fluid-CSF drainage pathways. CSF is formed in the specialized secretory tissue called the choroid plexus, which consists of epithelial cells, fenestrated capillaries and the highly vascularized stroma. Very little is currently known about the role played by the ventricles and the choroid plexus tissue in aging and Alzheimer’s disease (AD).

**Methods:**

In this study, we used our state-of-the-art proteomic platform, a liquid chromatography/mass spectrometry (LC-MS/MS) approach coupled with Tandem Mass Tag isobaric labeling to conduct a detailed unbiased proteomic analyses of autopsied tissue isolated from the walls of the inferior horn of the lateral ventricles in AD (77.2 ± 0.6 yrs), age-matched controls (77.0 ± 0.5 yrs), and nonagenarian cases (93.2 ± 1.1 yrs).

**Results:**

Ingenuity pathway analyses identified phagosome maturation, impaired tight-junction signaling, and glucose/mannose metabolism as top significantly regulated pathways in controls vs nonagenarians. In matched-control vs AD cases we identified alterations in mitochondrial bioenergetics, oxidative stress, remodeling of epithelia adherens junction, macrophage recruitment and phagocytosis, and cytoskeletal dynamics. Nonagenarian vs AD cases demonstrated augmentation of oxidative stress, changes in gluconeogenesis-glycolysis pathways, and cellular effects of choroidal smooth muscle cell vasodilation. Amyloid plaque score uniquely correlated with remodeling of epithelial adherens junctions, Fc *γ*-receptor mediated phagocytosis, and alterations in RhoA signaling. Braak staging was uniquely correlated with altered iron homeostasis, superoxide radical degradation and phagosome maturation.

**Conclusions:**

These changes provide novel insights to explain the compromise to the physiological properties and function of the ventricles/choroid plexus system in nonagenarian aging and AD pathogenesis. The pathways identified could provide new targets for therapeutic strategies to mitigate the divergent path towards AD.

## Background

The interstitial fluid (ISF) of the brain tissue and the cerebrospinal fluid (CSF) bathing the CNS are essential for providing a stable homeostatic and metabolic brain environment for neuronal function [1, 2]. They are also integral to maintaining adequate clearance of neuronal and glia waste products from the brain as a result of their ongoing metabolic activities. In late-onset Alzheimer’s disease (AD), it is becoming increasingly evident that inefficient brain clearance mechanisms may be the main driver for the accumulation of toxic proteinaceous aggregates [3–5]. For example, in pathological ageing potentially pathogenic Tau aggregates have been observed in and along the fluids of the brain, in periarterial/perivascular space, subependymal cells lining the walls of the ventricles, and subpial glial cells that form the glial limitans lining the brain parenchyma [6–14]. Cerebral amyloid angiopathy is also preferentially localized around leptomeningeal arteries between the pia and arachnoid compartments of the brain [11]. Thus, indicating that there may be abnormalities in the movement and clearance of solutes in brain fluids around these ventricular sites, and other CSF compartments that could contribute to tau/amyloid pathologies.

CSF is critical for brain clearance, it is produced from arterial blood by the choroid plexus located in the lateral and fourth ventricles, which consists of tufts of capillaries with an inner layer of fenestrated endothelial cells, an underlying thin layer of highly vascularized stroma, and an epithelial covering or lining of specialized apical ependymal cells with bulbous microvilli [1, 15]. CSF formation involves an active secretion process by these modified ependymal cells that forms the epithelial lining surrounding the plexus, involving pumps, co-transporters, antiporters, ion channels and aquaporins [1, 15]. The ventricular system has a variety of roles in brain physiology, but very little is currently known about the role(s) they play in ageing and AD pathogenesis, and how this may influence tau and amyloid proteinopathies.

Waste products (e.g. proteins) produced by neurons and glia can pass from the brain parenchyma by ISF convective bulk flow, moving through the ependymal ventricular lining and the glial limitans, to mix into the CSF for clearance into the venous circulation and lymphatic vessels around the cranial and spinal canal [1]. In the young adult CNS there is a volume of 160mls of CSF, and constant replenishment of this volume (4 times a day) is critical for the maintenance of extracellular fluid homeostasis [16]. This is accomplished by the modified choroidal ependymal cells which produces CSF at a rate of 0.3–0.6 ml per minute or 500–600 ml per day [16]. Balance of CSF formation and reabsorption is essential to the maintenance of CSF pressure, which has to be slightly greater than venous pressure in the dural sinuses to enable CSF absorption through arachnoid granulations/ villi (i.e. valve like structures) within the walls of the venous sinuses [16]. The epithelial lining of the ventricles also aids in the circulation of CSF through pulsations mediated by the motion of their specialized ciliated ependymal cells and the arterial hemodynamics in the plexus [2]. A tightly sealed barrier between the CSF and the blood, termed the blood-CSF barrier (BCSFB), is also formed by the tight junctions of these specialized choroid plexus ependymal cells which serves to inhibit paracellular diffusion of water-soluble molecules into the CSF. The BCSFB also serves as another site of waste clearance from the CSF into the blood. Ependymal cells forming the epithelial lining of the ventricles and their neighboring cells also have a prominent role as a secretory engine, producing growth factors and transporting hormones for neuroendocrine signaling [17]. They continuously regulate the chemical exchange between the CSF and the brain tissue, and the ionic environment (e.g. Ka+, Ca2+, HCO3-) of the brain fluids [1]. They are also one of the main sites for immune cell recruitment into the brain, and are involved in continually surveying the immunological status of the CSF [17].

In advanced ageing and/or AD cases, reports of alterations in BCSFB integrity and transporters accompanied by deficiencies in CSF production, enlargement of ventricular volume, increase in arachnoid villi resistance, and a reduction in the rate of lymphatic absorption have been documented [18–23]. If persistent, such alterations can have deleterious effects on brain physiology, leading to impaired efflux clearance of CSF waste products and influx of water soluble molecules directly into the brain, abnormalities in CSF production and circulation, irregular CSF pressure, edema formation, and altered recruitment of inflammatory cells into the CNS amongst a host of other outcomes. It remains unknown what chronic sequelae of events are responsible for driving the aforementioned age-related pathological changes observed in advanced ageing and AD.

Given the essential role of ependymal/epithelial cells, along with other neighboring cell types that forms the lining of the walls of the ventricles, in CSF fluid bio-functions, neuroendocrine function and immune cell recruitment, in this study we propose to explore the role that molecular abnormalities in this brain region may play in AD pathogenesis. Our hypothesis posits that the molecular integrity in the walls of brain ventricles of AD patients is significantly worse than controls and undemented cases, and this may contribute to the molecular consequences of AD pathogenesis (primarily tauopathy and amyloidopathy).

To address this, we will use our state-of-the-art unbiased proteomic platform, a liquid chromatography/mass spectrometry (LC-MS) approach coupled with Tandem Mass Tag isobaric labeling to conduct a detailed characterization and assessment of molecular changes in protein expression levels, and identify molecular pathways and biofunctions significantly altered in autopsied tissue isolated from the walls of the inferior horn of the lateral ventricles in AD (77.2 ± 0.6 yrs), age-matched controls (77.0 ± 0.5 yrs), and nonagenarian cases (93.2 ± 1.1 yrs). Unbiased proteomic analysis is a powerful tool which can provide a very expansive interrogation of the molecular response to diseases in a range of biomaterials. In this study we detail the unique proteomic profiles in isolated tissue from the superior part of the walls of the inferior horn of the lateral ventricles of AD and age-matched controls and nonagenarian patients with cognitive resilience.

## Methods

### Brain tissue

Human brain tissue samples from the superior part of the walls of the inferior horn of the lateral ventricles were obtained from the NIH NeuroBioBank at the Ican School of Medicine at Mount Sinai (New York, NY) in accordance with the institutional bioethics guidelines. All donors and their families gave written informed consent for autopsy and use of brain tissue for research purposes. As samples were obtained from deceased, de-identified, consenting individuals, no further ethical approval was required. On average, the autopsies were performed within 6–10 h after death. An overview of the demographics, clinical information and post-mortem variables of all brain donors used in this study is summarized in Table 1A-B. Neuropathological diagnosis of AD was determined using the Consortium to Establish a Registry for Alzheimer’s Disease (CERAD) diagnostic criteria [24] as well as the consensus recommendation for postmortem diagnosis of AD by the National Institute for Aging/Reagan Institute Working Group [25]. Neurofibrillary Tangle (NFT) distribution using the Braak staging method [26].

**Table 1:** (A) Top panel shows list of individual cases, their demographics, pathological score at autopsy and randomization of samples for Tandem Mass Tag isobaric 10-plex multiplexing. (B) Bottom panel shows mean estimates of relevant demographics, post-mortem interval (PMI) time, pathological score at autopsy for patients across the three different populations investigated

**A**								
**Cases**	**Disease**	**CDR score**	**Sex (M/F)**	**Mean plaque score**		**Braak-staging (I-VI)**	**TMT isobaric label**	**Plex group**
1	Healthy Controls	0	F	0		0	-128C	Plex A
2	Healthy Controls	0	F	0		I	-128N	Plex A
3	Healthy Controls	0	M	0		I	-129C	Plex A
4	Healthy Controls	0.5	M	0		II	-128C	Plex B
5	Healthy Controls	0	M	0.26		II	-128N	Plex B
6	Healthy Controls	0	M	0		0	-131 [ref]	Plex A, B, C
7	Healthy Controls	0.5	F	0		II	-129C	Plex B
8	Healthy Controls	0.5	M	0		I	-126	Plex C
9	Healthy Controls	0	F	0		0	-127C	Plex C
10	Nonagenerians	0	F	5.356		V	-127N	Plex B
11	Nonagenerians	0	F	1.944		IV	-126	Plex A
12	Nonagenerians	0.5	F	3.754		IV	-127C	Plex A
13	Nonagenerians	0	M	1.95		IV	-127N	Plex A
14	Nonagenerians	0.5	F	7.49		V	-126	Plex B
15	Nonagenerians	0.5	F	7.49		VI	-127C	Plex A
16	Alzheimer's Disease	4	M	29.76		VI	-129N	Plex A
17	Alzheimer's Disease	3	M	18.88		VI	-130C	Plex A
18	Alzheimer's Disease	2	F	19.12		VI	-130N	Plex A
19	Alzheimer's Disease	2	F	20.24		VI	-129C	Plex C
20	Alzheimer's Disease	1	M	6.72		VI	-128C	Plex C
21	Alzheimer's Disease	2	M	12.58		VI	-130C	Plex C
22	Alzheimer's Disease	3	F	9.17		VI	-127N	Plex C
23	Alzheimer's Disease	1	F	24.15		VI	-129N	Plex B
24	Alzheimer's Disease	3	F	20.48		VI	-130C	Plex B
25	Alzheimer's Disease	2	M	22.47		VI	-130N	Plex B
26	Alzheimer's Disease	3	F	31.96		VI	-128N	Plex C
**B**								
**Disease group**	**Mean PMI mins (±SEM)**	**Mean CDR score (±SEM)**	**Mean age Yrs (±SEM)**	**Male (%)**	**Female (%)**	**Mean plaque score (±SEM)**	**Braak-staging (±SEM)**	**Sample size (N)**
Healthy Control	601.6±94.6	0.17±0.08	77.2±0.6	55.6% (5/9)	44.4% (4/9)	0.03±0.03	1±0.3	9
Nonagenerians	448±110.7	0.25±0.11	93.2±1.1	16.7% (1/6)	83.3% (5/6)	4.66±1.03	4.67±0.3	6
Alzheimer's Disease	441.8±76.2	2.36±0.28	77.0±0.5	45.5% (5/11)	54.5% (5/11)	19.59±2.36	6±0.0	11

### Protein extraction

Frozen brain tissue blocks from the walls of the ventricles were homogenized in a 2.5fold weight per volume of LC/MS grade chilled water. 50 *u* l of homogenized tissue were added to a 50 *u* l aqueous buffer solution consisting of 2X PBS, 2X NaCl and cocktail of proteinase inhibitor. These samples were subsequently homogenized and centrifuged at 20,000 g for 15 min at 4 °C. To obtain nuclear and cytosolic fractions, the top supernatant layer (i.e. non-membrane fraction) was collected. The remaining pelleted samples were re-suspended in 1X volume of ice-cold methanol aqueous solution, and underwent centrifugation at 20,000 g (4 °C) for 15 min.

Subsequent precipitant was re-suspended in a 1X volume of ice-cold isopropanol and hexane (with a 2:1 dilution ratio), and centrifuged for 15 min at 20,000 g (4 °C). Resultant “membrane protein pellet fraction” was finally re-suspended in Triethylamonium bicarbonate (TEAB; 25 mM) and sodium deoxycholate (SDC; 0.5%). From the original supernatant (i.e. the initial non-membrane fraction) of the first centrifugation step above, samples were resuspended in ice-cold methanol at 0.5-fold volume, and centrifuged for 15 min at 20,000 g. The resultant “nuclear protein pellet fraction” were re-suspended in TEAB (25 mM) and SDC (0.5%). The supernatants of centrifuged samples from the last step were subsequently brought up in ice-cold methanol at 7-fold volume, followed by centrifugation for 15 min at 20,000 g, the resultant “cytosolic protein pellet fractions” were re-suspended in TEAB (25 mM) and SDC (0.5%). For the entire study, we used the membrane and cytosolic fractions.

### Tryptic digestion

A 1:100 enzyme-to-substrate ratio was used for our tryptic digestion. Briefly, sequencing grade porcine trypsin (Promega, Wisconsin) was added to 100 μg aliquots of depleted brain homogenate (i.e. cytosolic and membrane protein extracts) after reduction-alkylation procedure, at a final protein concentration of 1 μg/μl. Samples were subsequently incubated at 37 °C for 16 h. Ten microliters of digested samples were dried in a vacuum centrifuge for anhydrous TMT labeling described below.

### Tandem mass tag (TMT) labeling

A multiplexed isobaric labeling strategy (comprising of the 10-plex TMT labeling platform – ThermoScientific, Waltham, MA) was used to enable simultaneous identification and quantification of proteins from multiple biological samples. This enabled us to randomize all samples from all different groups for analyses within the same batch with the addition of an internal reference used in each batch. Experimenter was blinded to the identity of samples for each corresponding isobaric label tags. Briefly, twenty microliters of each TMT label was re-suspended in 25 mM TEAB in acetonitrile solution, and added to 20 μl of dried (digested) protein samples from each cases. Samples were incubated for 1 h under room temperature, with the reaction quenched with formic acid to a final concentration of 1% v/v. Labeled samples from each batch were pooled together based on their respective experimental batches and subsequently taken to dryness to remove acetonitrile prior to the SDC and TEAB cleanup step.

### Sample clean up

To remove residual SDC and TEAB from pooled samples, the dried TMT-labeled (protein) samples were re-suspended in 100 μl of 1% formic acid in LC/MS grade water, and subsequently centrifuged for 1 min at 20,000 g to remove SDC precipitant. Supernatants were collected into new tubes, and 400 μl of ethyl acetate was added, samples were then vortexed and centrifuged for 30 s at 20,000 g to partition residual SDC into the organic (upper) layer, with the latter discarded. This cycle was repeated on three occasions to ensure efficient removal of residual SDC, and the final lower phase was taken to dryness in a speed vacuum. Dried SDC cleaned samples were subsequently re-suspended in 100 μl of 0.1% formic acid. Pooled TMT-labeled samples were concentrated and de-salted using C18 reversed phase ZipTips following manufacturer’s protocol (Merk Millipore Ltd., Co. Cork, IRL). Final eluates of ZipTipping were re-suspended in 20 μl of 0.1% formic acid and transferred into an auto-sampler vial, for analyses using a nano-Ultra-Performance Liquid Chromatography (UPLC) MS on a Q-Exactive Orbitrap instrument (Thermo).

### Chromatography and mass spectrometry methods (LC-MS/MS)

We analyzed our protein samples by LC-MS/MS (Q-Exactive). Briefly, data dependent acquisition (DDA) settings for the MS experiments followed our previous work [27, 28]. DDA settings were as follows: full-scan MS resolution = 140,000 full width at half maximum at 200 m/z, full-scan range = 380–1250 m/z, isolation width = 1.2 m/z, higher energy C-trap dissociation relative collision energy = 29, a minimum m/z setting of 100 m/z was used for all MS^2^ spectra, MS^2^ resolution = 17,500, dynamic exclusion = 180 s, and a Top 15 high/low duty cycle was used for precursor ion selection. The narrow isolation window and the ultra-long gradient settings “were used to minimize the deleterious effects on quantitative accuracy that result from co-isolation of isobaric precursors without resorting to MS^3^-based methods” [27, 28].

### Data processing and statistical analysis of proteomics data

We used the PMi preview software to survey our amalgamated data-files and, to add other modifications to our search criteria, if deemed necessary. Preview results were used to choose the precursor and fragment ion mass tolerances (4-ppm, 0.02-Da, respectively) and dynamic modifications. We used the following settings to search the data using SEQUEST and BYONIC as the search algorithms, and Uniprot human database (FEB/2016). Dynamic modifications - Oxidation / + 15.995 Da (M), Methyl / + 14.016 Da (E), Deamidated / + 0.984 Da (N, Q), static modifications of TMT10plex / + 229.163 Da (N-Terminus, K), Carbamidomethyl + 57.021 (C). We only considered unique peptides for our final quantification. For SEQUEST, we used the Percolator feature of Proteome Discoverer, and for Byonic, we used the target-decoy feature, to set a false discovery rate (FDR) of 0.01. The peptides passing this stringent cutoff FDR rate were subsequently exported for data cleaning and statistical analysis. Proteins only underwent quantitative analysis if they were identified in at least half of the total number of plexes. A Shapiro-Wilk test for normality was assessed prior to statistical analyses. Raw ion counts were *ln* transformed and analyzed by One-way analyses of variance (ANOVA) (for 3 group comparison), t-test (2 group comparison) or spearman correlations to interrogate for significant proteins between the different groups. Significantly regulated proteins were subsequently uploaded into ingenuity pathway analyses (IPA) where molecules and pathways unique to each group comparison(s) were identified. The ratios were formed by first dividing all samples with the same reference samples from the control group (see Table 1A) used in the same plex, and subsequently log2 ratio values were generated by dividing log2 values between two groups of interest (i.e. control/AD or control/nonagenarians or AD/nonagenarians). We have deposited the mass spectrometry proteomics into the ProteomeXchange Consortium via the PRIDE partner repository [29]. Our datasets can be located with the unique identifier - PXD012059.

### IPA analysis

We uploaded all datasets of significantly modulated proteins from our group comparisons into the Ingenuity Pathway Analysis software (IPA, Ingenuity® Systems [30]) to map those significantly regulated proteins onto known networks of protein interactions in the knowledgebase. We also used the IPA knowledgebase to further ascertain the significantly regulated molecular pathways “Canonical pathways” and biological significance of AD, tau or amyloid dependent changes in protein expression from each experimental paradigm. For our core analysis settings, we used the following - Ingenuity Knowledge base as reference set, maximum number of 35 molecules per network, and maximum number of 25 networks for analysis. We only considered experimentally observed knowledge. We also controlled for species, data sources, tissue type/cell lines at the time of analysis in IPA. Our core analysis identified canonical pathways shown to be significantly modulated in response to AD, tau or amyloid pathogenesis as a result of significant modulation of proteins represented in those pathways. The statistical significance of the association between the uploaded dataset and the identified canonical pathways were measured using two methods: 1) Ratio of the number of molecules from the data set that map to a canonical pathway divided by the total number of molecules in that canonical pathway knowledgebase in IPA is displayed. 2) Fisher’s exact test, to calculate a *p*-value determining the probability that the association between the proteins in the dataset and the canonical pathway is explained by chance alone. *P*-values lower than 0.01 were only considered significant in these studies. Upstream regulator analysis in IPA was used to predict the upstream master regulators in our proteomic dataset using the Ingenuity® Knowledge Base. An overlap *P* values was determined based on analyses of the significant overlap between proteins (and their related genes) in our dataset and known targets regulated by the Upstream master regulator.

### Western blotting analyses

Immunoblotting analyses was conducted on tissue homogenates prepared for proteomic analyses above. Briefly, homogenized samples resuspended in TEAB (25 mM) and SDC (0.5%) were first denatured at 95 °C by boiling in Laemmli buffer (Bio-Rad; Hercules, CA) with reducing agent. Samples were then resolved on stain free 4 to 20% gradient polyacrylamide criterion gels (Bio-Rad; Hercules, CA). After electrotransferring, polyvinylidene difluoride (PVDF) membranes were blocked in 5% milk (made in Tris-buffered saline - TBS) and subsequently immunoprobed with primary antibodies overnight (Neurofilament medium chain polypeptide [NEFM] and Neurofilament medium chain polypeptide [NF-H] - ThermoScientific, Waltham MA; Superoxide dismutase [SOD1] – Abcam, Cambridge MA). Washed membranes were then probed with respective horseradish peroxidase (HRP)-linked anti-mouse and anti-rabbit secondary antibodies (Santa Cruz; Dallas, TX). Immunoblots were analyzed by using the total protein quantitation of stain free gels to obtain signal intensity densitometry ratios quantified by chemiluminescence imaging using the ChemiDocTM XRS (Bio-Rad; Hercules, CA).

## Results

### Demographics and clinical background of patient population

In this study, we used 26 total brain specimens from the superior part of the inferior horn of the lateral ventricles obtained from controls (9 cases), aged-matched controls (6 cases) and nonagenarians(11 cases) – Table 1A-B. On average both the nonagenarian (448 ± 110.07) and AD (441 ± 76.2) groups had a similar post-mortem interval time; with the control group were 25–27% higher compared to the nonagenarian and AD groups. Control group were 77.2 ± 0.6 yrs. and AD group 77.2 ± 0.5 yrs.; while the nonagenarian group were 93.2 ± 1.1 yrs.

Each group had a mixed gender, with 44.4, 83.3 and 54.5% females observed in control-AD, nonagenarian and AD groups respectively. Controls (0.17 ± 0.08) and nonagenarian (0.25 ± 0.11) groups showed relatively similar CDR score; whilst AD group showed significantly higher CDR scores at (2.36 ± 0.28) as expected. Mean amyloid plaque score in control, nonagenarian and AD groups were 0.03 ± 0.03, 4.66 ± 1.03 and 19.59 ± 2.36 respectively. Braak staging score in control, nonagenarian and AD groups were 1.0 ± 0.3, 4.67 ± 0.03 and 6.0 ± 0.0 respectively.

### Proteomic profiles and the cell type distribution of the superior part of the inferior horn of the lateral ventricles from autopsy tissue of control, AD and nonagenarian patients

**Fig. 1:**
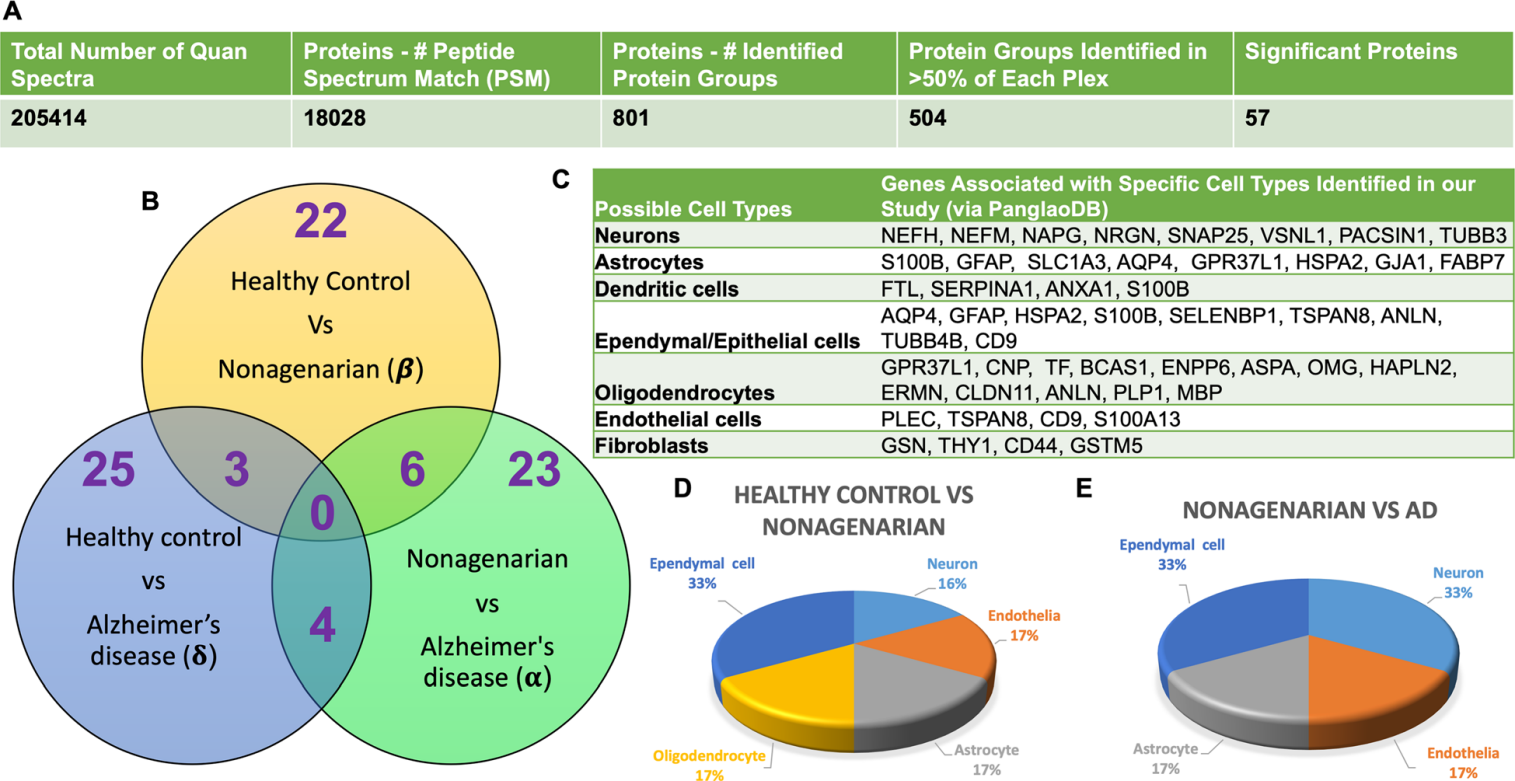
Summary of liquid chromatography/mass spectrometry (LC/MS) and proteomic analyses of tissue from the inferior horn of the lateral ventricles in Alzheimer’s disease (AD) patients, their aged-matched controls and nonagenarian controls. **a** Table shows identified total number of quantified spectra, peptide spectrum matches, non-redundant master protein groups, number of master protein groups identified in 50% of all plexes and used for analyses. **b** Venn diagram shows significantly regulated proteins in the comparisons between AD patients, their aged-matched controls and nonagenarian controls. Green (α) represents nonagenarians vs AD; Yellow (β) represents controls vs nonagenarians; Blue (δ) represents AD-controls vs AD. **c** Table shows gene IDs (obtained via the PanglaoDB database) associated with specific cell types identified from our proteomics study of the lateral ventricle. **d** and **e** Pie Charts show cell types with significant changes in proteins/genes associated with those cells (see C), expressed as a percentage between controls vs nonagenarian **(d)** and nonagenarians vs AD **(e)** (due to the minimal changes in identifiable genes/proteins associated with these cells types between control vs AD we have not included that pie chart herein)

A 10multiplex TMT isobaric tag approach was used to study the proteomic profiles in the superior part of the inferior horn of the lateral ventricles of control, AD and nonagenarian patients. In this study, a total of 18,028 total peptide spectrum matches, 801 non-redundant master protein groups, 504 of these master protein groups were identified within 50% of each plex and groups (Fig. 1a- *table*). A one-way ANOVA approach was used to analyze the master proteins to identify changes in unique and common proteins significantly changing between control vs AD vs nonagenarian patients.

**Fig. 2:**
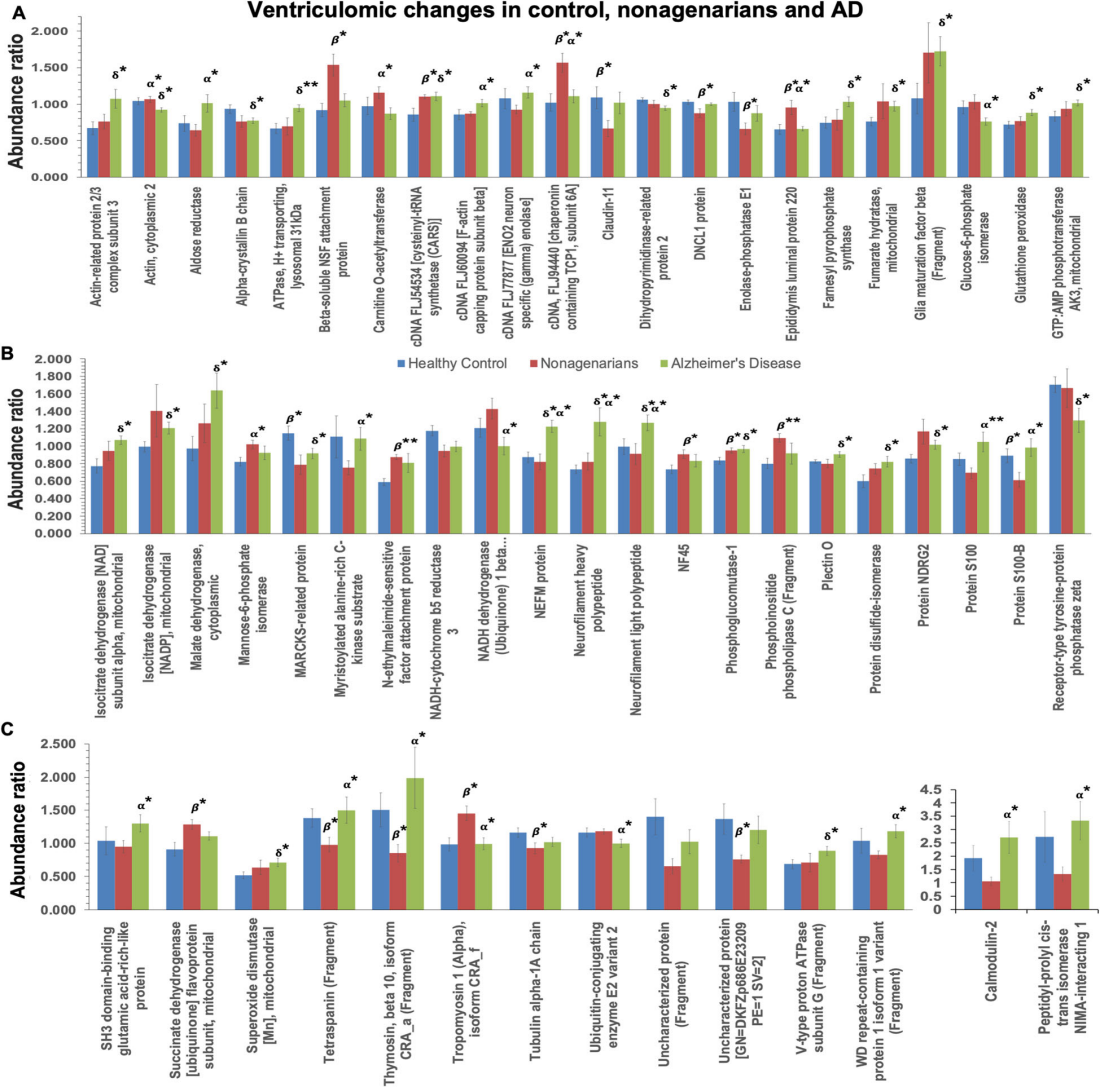
Graph showing significantly modulated proteins (their abundance ratio) in the inferior horn of the lateral ventricles AD patients, their aged-matched controls and nonagenarians. Asterisks denote: *P < 0.05; **P < 0.01; ***P < 0.001. *P* value represents FDR adjusted *p* value after ANOVA (/Tukeys HSD posthoc test) and Benjamin Hochberg correction. ***δ*** - control vs AD; ***β*** - control vs Nonagenarians; ***α*** - Nonagenarians vs AD

Statistical analyses using one-way ANOVA after Benjamin Hochberg correction identified 22 proteins changing in the control vs nonagenarian patients, 25 proteins changing in the control vs AD patients, and 23 proteins changing in nonagenarian vs AD patient’s (Fig. 1b – *see Venn diagram*). In the control vs nonagenarian and control vs AD groups, 3 common proteins were identified to be significantly altered. In the nonagenarian vs AD and control vs AD groups, 4 common proteins were identified to be significantly altered. In the control vs nonagenarian and nonagenarian vs AD groups, 6 common proteins were identified to be significantly altered. See Table 2 and Fig. 2 (graph representation) for the list of significantly altered proteins between the three different groups.

**Table 2:**
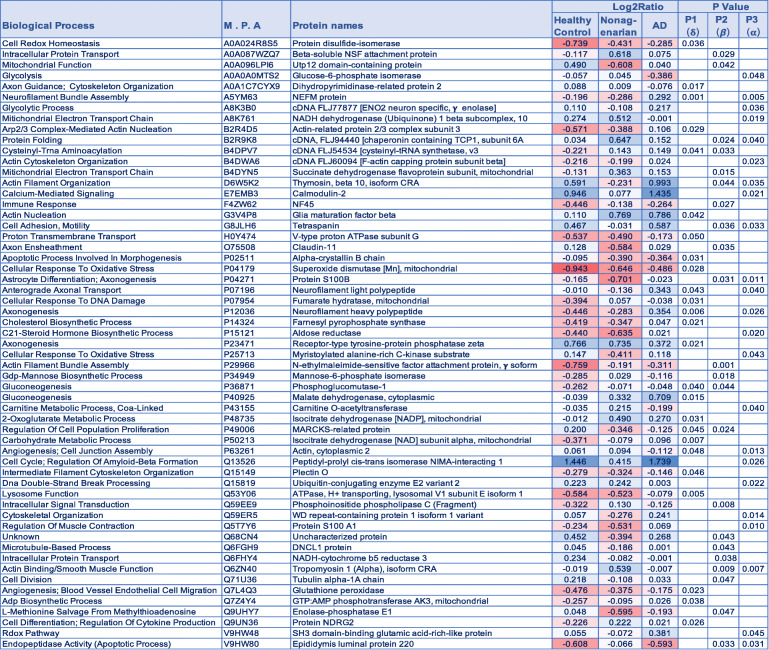
List of significantly modulated proteins in the inferior horn of the lateral ventricles of AD patients, their aged-matched controls and nonagenarians. *P* value represents FDR adjusted *p* value after ANOVA and Benjamini Hochberg correction. Red – downregulated across group; Blue – upregulated across group. M.P.A – Master Protein Accession Number

We interrogated the cell types observed in our proteomic analyses by identifying proteins (or their related gene IDs) associated with brain specific cell types in the PanglaoDB database. A list of gene IDs identified in our proteomic runs and matched to their potential cell types is shown in Fig. 1c. We identified 8 markers associated with neurons, 8 markers associated with astrocytes, 4 markers linked to dendritic cells, 9 markers associated with ependymal/epithelial cells, 13 markers linked to oligodendrocytes, 4 markers associated with brain vascular endothelial cells, and 4 markers linked to fibroblasts/immune cells. A few of these markers overlapped with a maximum of two cell types. Although not an indication of cell density, this gives an indication of the variety of cell types in our tissue samples. We additionally interrogated the number of significant proteins changing between the different groups that was associated with the variety of these cell types, and expressed this as a percentage in Fig. 1d-e. We observed that most of the significant changes in proteins observed between controls vs nonagenarian were associated with ependymal cells (33%); neurons, endothelia, astrocytes and oligodendrocytes each had a 16–17% involvement (Fig. 1d). In nonagenarians vs AD cases most of the significant changes in proteins were associated with ependymal cells (33%) and neurons (33%), while astrocytes and endothelial cells each had a 17% involvement (Fig. 1e). Due to the minimal changes in identifiable proteins specifically associated with these cells types in our significant list of proteins from control vs AD we could not obtain similar analyses to infer cell specific changes for this particular comparison.

### Disease, biofunctions, canonical pathways and upstream regulators modulated in the superior part of the inferior horn of the lateral ventricles in autopsy tissue from control, AD and nonagenarian patients

A list of 35 diseases and biofunctions modulated in the superior part of the inferior horn of the lateral ventricles of control vs AD vs nonagenarian patients is shown in Supplementary Table T1. Some of these diseases and biofunctions include alteration in formation of microtubules and cytoskeleton, vascularization, cell death/apoptosis, proliferation of neuroglia, synthesis of nitric oxide, cell death of connective tissue cell, microtubule dynamics, migration and invasion of cells. Ingenuity pathway analyses (IPA) identified 9 pathways significantly impacted between control vs nonagenarian group, 12 pathways significantly impacted between control vs AD group and 5 pathways significantly impacted between nonagenarian vs AD group (Table 3). In the control vs nonagenarian group, the top 3 pathways significantly regulated were phagosome maturation, tight-junction signaling and D-mannose degradation. In the control vs AD group, the top 3 pathways significantly regulated were Amyolateral sclerosis signaling, TCA cycle II, and remodeling of epithelial adherens junctions. In the nonagenarian vs AD group, the top 3 pathways significantly regulated were Amyolateral sclerosis signaling, gluconeogenesis I, and glycolysis.

**Table 3:**
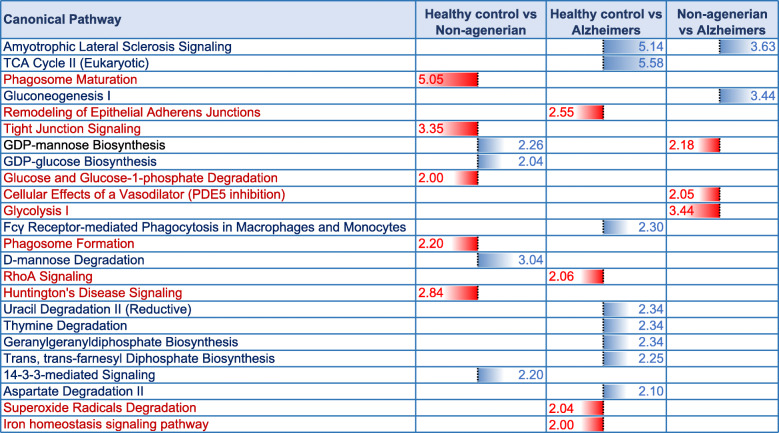
Canonical pathways modulated in the inferior horn of the lateral ventricles of AD patients, their aged-matched controls and nonagenarians. Identified canonical pathways generated from the list of significantly modulated proteins across three groups of interests using Ingenuity pathway analyses (IPA). FDR adjusted *P* value after Fischer’s test and Benjamin Hochberg correction following uploading of significantly regulated proteins into IPA (significant cut-off set at 0.01). Control vs nonagenarians (9 pathways identified), control vs AD (12 pathways identified), control vs nonagenarians (5 pathways identified). Blue represents upregulated pathways and Red represents downregulated pathways

**Fig. 3:**
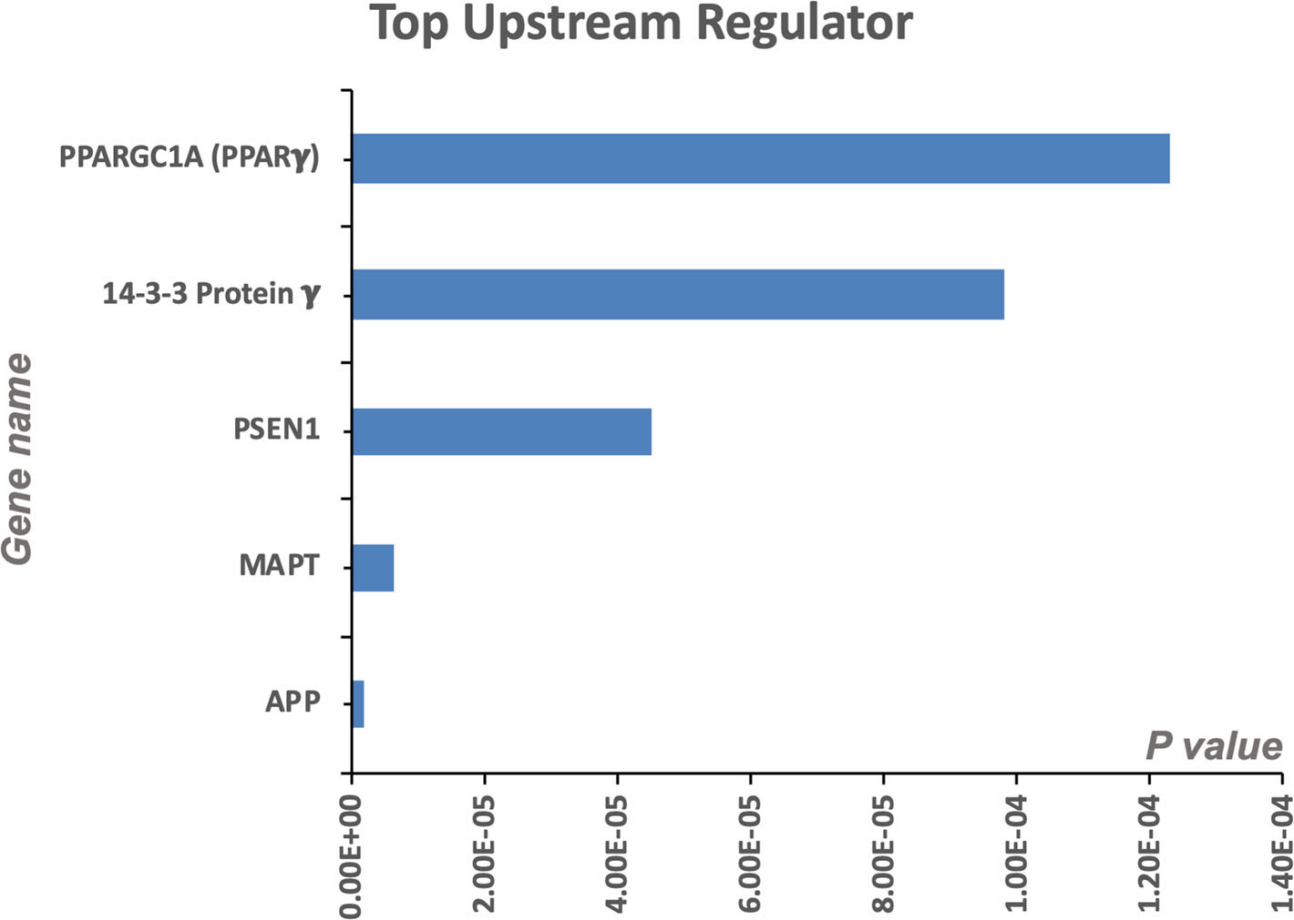
Top 5 Upstream Regulators in the inferior horn of the lateral ventricles. PPARGC1A (PPARG Coactivator 1 Alpha), 14–3-3 protein gamma (or Protein kinase C inhibitor protein 1), PSEN1 (Presenilin 1), MAPT (Microtubule associated protein tau gene), and APP (amyloid precursor protein) were the top five upstream regulators identified by IPA. *P* values were determined based on analyses of the significant overlap between genes/proteins in our dataset and known targets regulated by the Upstream master regulator. All 5 upstream regulators were observed from our 3 combinations of Control vs AD, control vs nonagenarian and nonagenarian vs AD cases

We interrogated Top 5 Upstream Regulators of the significant proteins identified in our analyses across all three group comparisons (Fig. 3). IPA analyses identified a significant overlap in our dataset and known targets regulated by these five Upstream regulators, namely (in order of the level of significance) - PPARGC1A (PPARG Coactivator 1 Alpha a transcriptional co-activator), 14–3-3 protein gamma (or Protein kinase C inhibitor protein 1 an adapter protein involved in a variety of signal transduction pathways), PSEN1 (Presenilin 1, a proteolytic subunit of γ-secretase involved in cleavage of many transmembrane proteins such as APP), MAPT (microtubule associated protein tau gene involved in the production of tau cytoskeletal proteins), and APP (amyloid precursor protein, a transmembrane enzyme cleaved into soluble APP and amyloid beta peptide). All 5 upstream regulators were observed from our comparisons between Control vs AD, control vs nonagenarian and nonagenarian vs AD cases.

### Proteomic profiles of the superior part of the inferior horn of the lateral ventricles in autopsy tissue stratified to amyloid plaque score and Braak staging

**Fig. 4:**
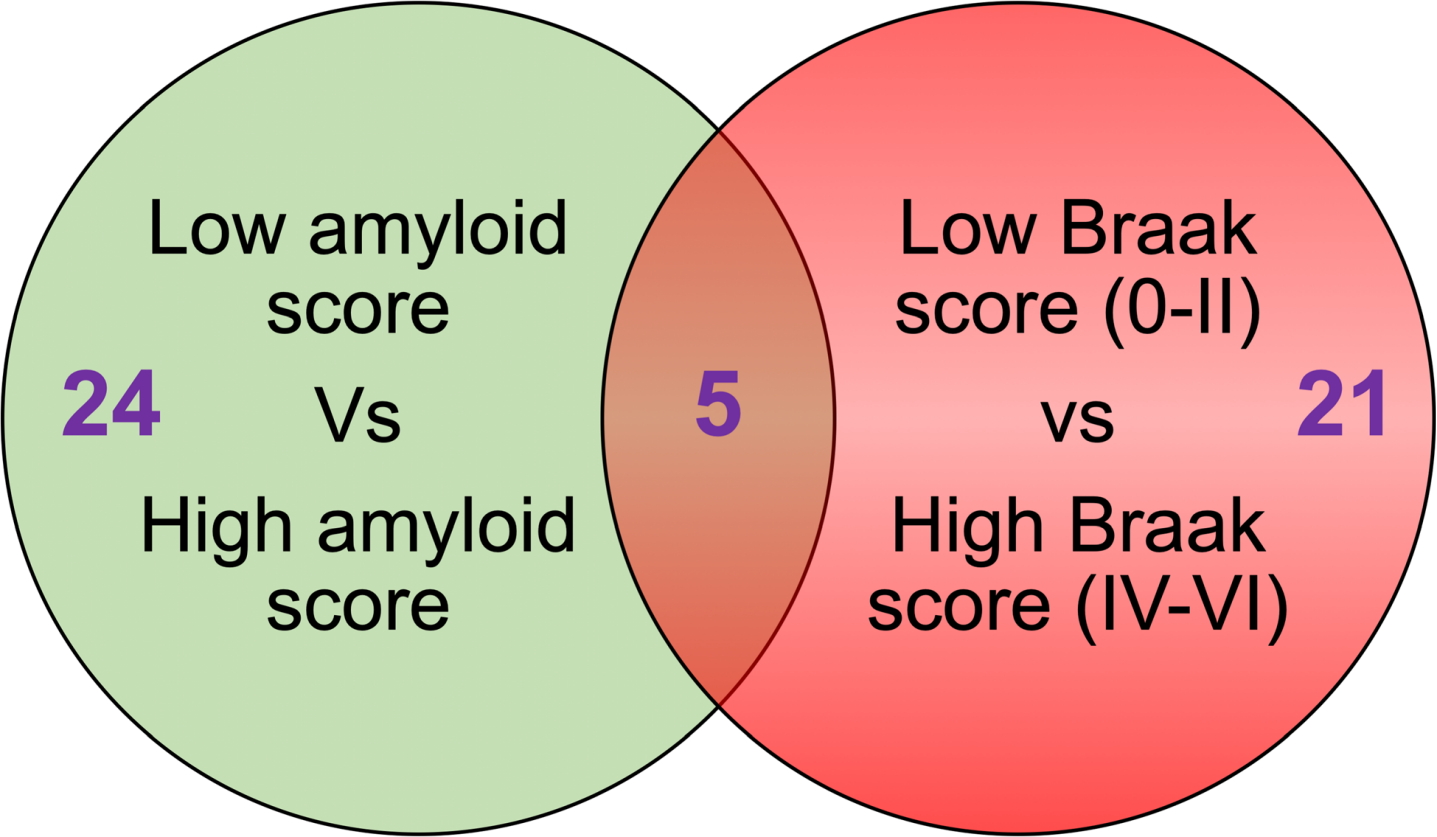
Venn diagram showing list of significant proteins in the inferior horn of the lateral ventricles based on stratification with amyloid plaque score (low < 10 vs high > 10) and Braak staging (0-II vs IV-VI)

In the low amyloid plaque score (< 10) vs high amyloid plaque score (> 10) we identified 24 significant proteins (Fig. 4 – Venn diagram). In the early Braak staging score (0-II) vs late Braak staging score (IV-VI) we identified 21 significant proteins (Fig. 3 – Venn diagram). There were 5 common proteins significantly changing in both stratifying groups (*see red highlight* in Table 4 and 5). For a list of significantly altered proteins stratified to amyloid plaque score and Braak staging groups please see Table 4 and 5.

**Table 4:**
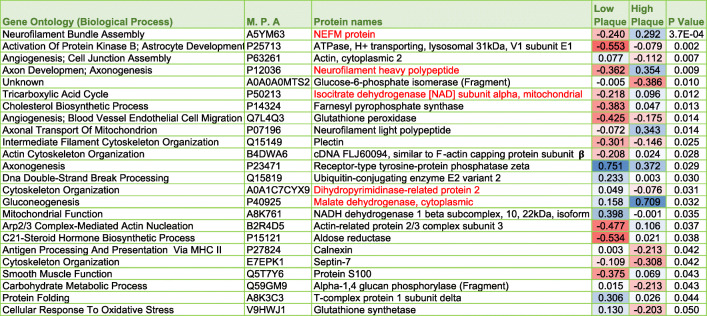
List of significantly modulated proteins in the inferior horn of the lateral ventricles based on stratification with amyloid plaque score (low < 10 vs high > 10). *P* value represents FDR adjusted *p* value after ANOVA and Benjamin Hochberg correction. Blue box – upregulated across group; Red box – downregulated across group. Red highlighted text shows significant proteins overlapping after stratifying with amyloid plaque scoring and Braak staging (see Table 5)

**Table 5:**
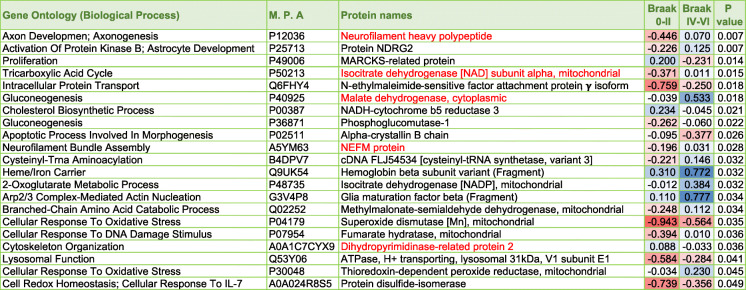
List of significantly modulated proteins in the inferior horn of the lateral ventricles based on stratification with Braak staging (0-II vs IV-VI). *P* value represents FDR adjusted *p* value after ANOVA and Benjamin Hochberg correction. Blue box – upregulated across group; Red box – downregulated across group. Red highlighted text shows significant proteins overlapping after stratifying with Braak staging and amyloid plaque scoring (see Table 4)

### Disease, biofunctions and canonical pathways modulated in the superior part of the inferior horn of the lateral ventricles in autopsy tissue stratified to amyloid plaque score and Braak staging

A list of 21 diseases and biofunctions modulated in the superior part of the inferior horn of the lateral ventricles of patients stratified to low amyloid plaque score (< 10), high amyloid plaque score (> 10), early Braak staging (0-II) and late Braak staging (IV-VI) is shown in Supplementary Table T2. Some of these diseases and biofunctions include cell death, necrosis, microtubule dynamics, cell viability, formation of cytoskeleton and microtubule, accumulation of filaments, apoptosis of muscle cells, quantity of cell protrusions, and transport of molecules. Ingenuity pathway analyses (IPA) identified 11 pathways significantly impacted between early Braak staging (0-II) vs late Braak staging (IV-VI), 14 pathways significantly impacted between low amyloid plaque score (< 10) vs high amyloid plaque score (> 10) (Table 6). In the group comparisons stratified to Braak staging, the top 3 pathways significantly regulated were TCA cycle II, mitochondrial dysfunction and *β*-alanine degradation I. In the group comparisons stratified to amyloid plaque staging, the top 3 pathways significantly regulated were Amyolateral sclerosis signaling, TCA cycle II, and RhoA signaling.

**Table 6:**
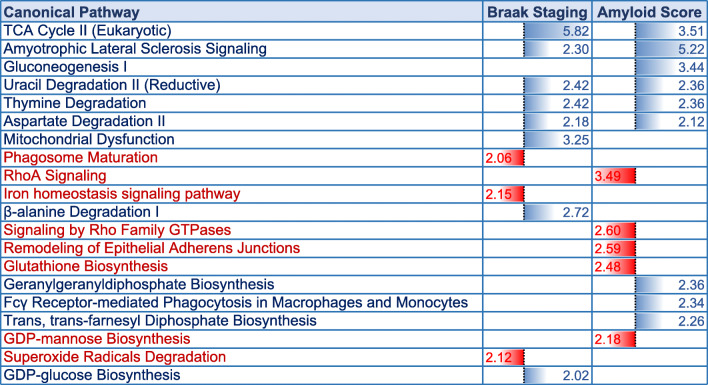
Canonical pathways modulated in the inferior horn of the lateral ventricles after stratification for Braak staging and amyloid plaque score. Identified canonical pathways generated from the list of significantly modulated proteins after stratifying for low vs high amyloid plaque score (low < 10 vs high > 10) and early vs late Braak staging (0-II vs IV-VI) using Ingenuity pathway analyses (IPA). FDR adjusted *P* value after Fischer’s test and Benjamin Hochberg correction following uploading of significantly regulated proteins into IPA (significant cut-off set at 0.01). Early vs late Braak staging (11 pathways identified), low vs high amyloid plaque score (14 pathways identified). Blue represents upregulated pathways and Red represents downregulated pathways

### Validation of proteins identified from proteomic studies

Three proteins (Neurofilament medium chain polypeptide [NEFM], Neurofilament medium chain polypeptide [NF-H], Superoxide dismutase [SOD1]) were selected from the list of most significantly regulated proteins across different groups from our proteomic analyses for validation of our LC/MS-MS data using antibody based methods. NEFM and NF-H and SOD1 showed a similar trend in the direction of changes in control, nonagenarian and AD groups using western blotting technique compared to findings observed from our proteomic LC/MS-MS analyses in the same tissue samples (see Supplementary Figure S1:AA’-EE’).

## Discussion

Very few studies have explored the pathological changes to ependymal cells and specialized choroid plexus epithelial cells that line the ventricles in the pathogenesis of AD. To address this, we employed our state-of-the-art unbiased proteomic platform to conduct a detailed unbiased characterization of changes in protein expression levels, and molecular pathways significantly altered from autopsy tissue obtained from the walls of the ventricles (i.e. the superior part of the inferior horns of the lateral ventricles) in AD cases, their age-matched controls, and a rare population of non-demented nonagenarians. Multiciliated ependymal cells predominantly comprise the epithelial layer lining the walls of the lateral ventricles, supported by a periventricular niche of different cell types. Our analyses revealed unique changes in proteins and molecular pathways that could contribute to AD pathogenesis and successful ageing.

### Ventriculomics changes in AD patients

The lateral ventricles are the two largest cavities of the ventricular CSF system within the human brain. The inferior horn is the largest horn of the lateral ventricles, it sits around the temporal lobe and its superomedial portion is one of the localized sites of the choroid plexus. One of the main roles of this specialized ventricular structure, is to monitor and maintain both biochemical and cellular homeostasis of the CNS environment. This region is also a primary route for exchange between the blood and the CSF, formed by tight junctions of specialized ependyma cells at the blood-CSF barrier (BCSFB). These barrier functions are maintained by the expression of numerous membrane transport systems such as ATP-binding cassette transporters, solute carrier families and peptide transporters allowing the directed transport of water, ions, nutrients, and biologically active compounds into the CSF from the blood [31]. The fenestrated capillaries of the BCSFB also serves as an important site and route of clearance (eventually into the blood) for brain derived molecules originating from ISF bulk flow that mix with the CSF after passing through ependymal cells lining the walls of the ventricles and glia limitans. Ependyma cells lining the walls of the ventricles also contribute to the production of up to 600 ml of CSF per day at a rate of 0.3–0.6 ml per minute [32]. They are involved in the circulation of 160mls of (adult) CSF through pulsations mediated by the motion of their ciliated and bulbous microvilli in combination with the arterial hemodynamics in the plexus [32]. Trafficking of immune sentinel cells (such as macrophages or dendritic cells) also occur across the ependymal cells lining the walls of the ventricles [33]. Ependymal cells also have a high protein secretory capacity, that releases transport proteins, collagen subunits and other cell matrix proteins, proteases, cytokines, neuropeptides/hormones and critical neurotrophic factors involved in neuroendocrine function, immune homeostasis and neuronal support/proliferation [34–36].

In pathological ageing (e.g. AD), the brain has been reported to demonstrate evidence of ependymal/epithelial atrophy, thickening of the basement membrane, stroma fibrosis, alterations in BCSFB integrity, deficiencies in CSF production and composition, enlarged ventricular volume, increased arachnoid villi resistance, and reduced rate of lymphatic absorption [18–23]. Moreover, toxic proteins in pathological ageing (such as amyloid-ß and hyperphosphorylated aggregated tau) feature around periventricular regions next to the cerebrospinal fluids of the brain (in and around subependymal cells lining the walls of the ventricles and subpial glial cells), indicating abnormalities in the movement and clearance of solutes around these ventricular sites [6–14]. The molecular sequelae of events driving these age-related pathological changes remains elusive. Studying the molecular response and proteomic profiles of these unique cell types and stromal responses along the walls of the ventricles may thus provide insights into the age-related neurodegenerative disease state.

Herein, we interrogated autopsy tissue from the walls of the lateral ventricles of AD and age-matched control patients using an unbiased proteomic analyses, and revealed a significant increase in 25 proteins. IPA analyses identified molecular pathways significantly altered between these two groups, consisting of changes in aspects of amyotrophic lateral sclerosis signaling, superoxide radical degradation, tricarboxylic acid cycle (TCA) cycle and disruptions in several energy metabolic pathways, remodeling of the epithelial adherens junctions, Fc *γ* receptor mediated phagocytosis in macrophages and monocytes, and RhoA signaling which is implicated in cytoskeletal dynamics, transcription, cell cycle maintenance and cell development. Some of these effects correlated with the staging of pathology, being more prominent in Braak stage IV-VI vs Braak Stage I-III. Our observed changes ostensibly indicate molecular abnormalities in the walls of the lateral ventricles typified by alterations in mitochondrial bioenergetics, oxidative stress, impaired barrier (BCSFB) integrity, recruitment of macrophages, and alterations in the cytoskeletal organization of cells. To confirm our LC-MS/MS data we validated a few proteins from our proteomic profiles using antibody based methods, which showed similar trends and corroborated our data.

To date, very few studies have utilized an omic platform to explore the molecular changes to tissue from the walls of the ventricles during AD pathogenesis. Most studies appear to have focused primarily on the choroid plexus using gene arrays. For example, a transcriptomic study used an Agilent platform with common reference design, to perform a large scale gene expression analysis and functional annotation of autopsied Choroid Plexus tissue from patients with Braak stages 0-I and V-VI, including their age matched controls [37]. Isolated RNA was collected from laser microdissected Choroid Plexus Epithelium cells on cryo-sections. Data revealed specific cellular changes attributed to increased oxidative stress, such as the unfolded protein response, Eukaryotic Initiation Factor 2 (E1F2) and Nuclear factor erythroid 2-related factor 2 (NRF2) signaling and the protein ubiquitin pathway. They also noted that BCSFB barrier and paracellular transport may become more permeable in AD, due to the downregulation of tight junction protein, Claudin-5. The findings also predicted loss of choroid plexus epithelium mediated macrophage recruitment, and down-regulation of (anti-oxidant) glutathione mediated detoxification pathway and the urea cycle in AD tissue, suggesting that the choroid plexus epithelium sink action may be impaired in AD.

In another study, utilizing transcriptome-wide Affymetrix microarrays of the extracted lateral ventricular choroid plexus tissue of AD Braak stage III–VI vs control cases, significant upregulation in genes related to metabolic and immune-related pathways including acute phase response, cytokine, cell adhesion, interferons, Janus Kinase and Signal transducer and activator of transcription protein (JAK-STAT) and Mammalian target of rapamycin (mTOR) were observed. Downregulated genes included tight-junction adherens (claudin-5), and genes related to amino acid (methionine) degradation and protein translation [38].

Another recent study utilizing the Brown-Merck Gene Expression Omnibus database (CP transcripts) to interrogate genes in AD vs control choroid plexus tissue also revealed changes in ion transporters (e.g. solute carrier SLC4A5) and related enzymes (e.g. carbonic anhydrase CA4), decreased expression of tight junction protein, claudin-5 involved and mitochondrial ATP synthesis (e.g. adenosine triphosphate ATP5L), increased expression of pro-inflammatory mediator, interleukin 1 receptor like 1 (IL1RL1) and amyloid precursor protein (APBA3), [39]. Further supporting evidence of disruptions to CSF production, solute transport at the blood–CSF interface, brain inflammatory status, and mitochondrial bioenergetics in AD.

Additionally, in a preceding proteomic study, using the less stringent 2D difference gel electrophoresis method to interrogate choroid plexus tissue of patients at different stages of AD, authors reported dysfunction in cytoskeletal integrity as one of the main pathogenic events in AD, corroborating our own LC-MS/MS findings [40]. They particularly noted a significant reduction in moesin at early stages of AD pathology. Moesin is a member of the ezrin/radin/ moesin family of actin binding proteins, which plays a crucial role in the maintenance of cell-to-cell adhesion, cell shape and motility, and membrane trafficking in choroidal epithelial cells and ciliated ependymal cells lining the walls of the ventricles. Their activity is regulated by a RhoA dependent signaling, which was also significantly altered in our proteomic study. Maintenance of cytoskeletal integrity is crucial to the function of the choroid plexus as it is need for the assembly and positioning of specialized intracellular junctions between epithelial cells, needed to maintain epithelia adherens junction formation and barrier characteristics of the BCSFB. This may further serve to explain why we and others [37–39] observed alterations in adherens junction proteins and BCSFB integrity. Dynamic changes in the cytoskeleton also serves an important factor in the cell cycle maintenance and progression of the choroid plexus epithelial cells and ependymal cells lining the walls of the inferior horn of the ventricles, needed to maintain adequate cell numbers required for efficient production and circulation of CSF. In AD, there is a noted reduction in CSF production rate [41], reduced water influx (ratio) into the CSF [42–44], and also an elevated CSF pressure [45]. These CSF changes can have significant effect on secretion, circulation, absorption and drainage/clearance processes in the brain of AD patients. Indeed, studies have shown that clearance rates of Aß are slower in individuals with AD compared to age-matched controls [46], while tau production rate in the CSF has also been shown to positively correlate with amyloidosis in AD [47] .

Our further search of the literature also identified a consistent theme from other studies utilizing (biased) proteomic and antibody-based analytical methods, which corroborated with our studies and other unbiased omic analyses mentioned above [37–39]. This involved a prominent interplay between defects in energy metabolism and oxidative stress. For example mitochondrial enzyme defects typified by reductions in cytochrome *c* oxidase activity and an increase in the density of cytochrome c oxidase-deficient choroidal epithelial cells was observed in the ventricular tissue/choroid plexus epithelium of AD patients compared to normal aged matched patients [48]. Impairment in the activity and assembly of mitochondrial respiratory chain complexes I and IV, and upregulation of mitochondrial stress related (chaperone) proteins (e.g. heat shock protein (Hsp)60 and (Hsp)90) were also observed in the choroid plexus/ventricles following exposure to Aβ [49]. Dysfunction in mitochondrial bioenergetics in the brain parenchyma is one of the earliest deficits observed in AD brains [50]. The ependymal cells lining the ventricles have a high metabolic demand owing to their high secretory capacity and transport systems, thus subtle changes in their mitochondrial bioenergetics can diminish the efficiency of their essential cellular functions.

Damage to mitochondria can also lead to the increased production of mitochondrial reactive oxygen/nitrogen species which can trigger oxidative stress and subsequently oxidative and nitrative damage to proteins. We observed an alteration in the degradation of superoxide free radical species in our studies. Upregulation of nitric oxide (NO) production has also been reported within the choroid plexus of AD patients, associated with Aβ deposits [51]. This increase in NO plays a negative feedback role in Aβ-induced mitochondrial dysfunction [52], as the interaction between NO and cytochrome *c* oxidase regulates mitochondrial production of reactive oxygen species. Moreover, reduction in oxidant-induced Nrf2-regulated gene products such as anti-oxidant Heme oxygenase-1 (HO1), have likewise been observed in the choroid plexus/ventricles of AD brains, in agreement with low HO-1 protein levels in the CSF [53, 54]. Anti-oxidant enzyme, aldehyde dehydrogenase (ALDH), has also been shown to reduce with the extent of Braak staging (V-VII vs I-II), despite an initial increase in early stages (Braak I-II vs control) of AD pathogenesis [40].

Studies have also confirmed oxidative damage to key proteins in the choroid plexus compared to other brain regions at early and/or late stages of AD. This includes, *signaling protein -* 14-3-3, *actin regulatory protein* - tropomyosin, and *lipid carrier* - apolipoprotein A-II [40, 55]. Oxidative damage to these proteins may alter protein-protein interactions, protein folding and protein kinase activity, potentially impairing cell signaling, mitochondrial function, cilia motility, lipid transport and metabolism [55].

Another hallmark feature observed in our study is the alteration in the recruitment of inflammatory cells, and a pro-inflammatory state of cells lining the ventricular tissue in AD brains. To corroborate these findings, in the aforementioned transcriptomic study by Kant et al. (2018) [39], a significant increase in interleukin-1 receptor (IL1R) and IL1RL1 was noted in AD vs control cases. IL1R/IL1RL1 signaling are involved in promoting acute and chronic inflammation, and could provide a chemotactic gradient to recruit circulating blood borne monocytes, as this region can serve as an interface between the brain-CSF and the circulation [56]. Similar changes were also corroborated by other transcriptomic studies in human choroid plexus tissue typified by altered choroid plexus mediated microphage recruitment [37] and augmented immune-related pathways including acute phase response, cytokine, and interferon signaling [38]. This is also supported by studies in chronic advanced aged mice, where multiorgan genome-wide analysis reported a type I interferon (IFN-I)-dependent gene expression profile and signature in the choroid plexus [57]. This altered inflammatory state in the choroid plexus was shown to partially drive cognitive dysfunction and deficiency in hippocampal neurogenesis as demonstrated by the restorative effects of blocking IFN-I signaling [57]. In our study we observed a significant increase in Fc *γ* receptor mediated phagocytosis in macrophages and monocytes in the ventricular tissue of AD compared to control subjects. Thus it appears that during AD pathogenesis, there could be an alteration in the trafficking of blood derived macrophage cells into the ventricular/choroid plexus tissue, which could compromise and lead to a pro-inflammatory environment.

A major point of consideration for our study is the timeline of major pathogenic events in AD and their relationship to changes observed in the lateral ventricles. Studies have reported high levels of amyloid beta, and tau accumulation (observed as argyrophilic filaments, curly fibers, and tangle pathology), in the choroid plexus of AD patients compared to age-matched non-demented subjects [58–63]. Thus suggesting a direct relationship between these pathogenic proteins and the development of functional and structural deterioration [58]. It remains undetermined whether amyloidosis and tauopathy in AD, can occur as a direct consequence of early choroid plexus/ventricular system deterioration, or whether tau/amyloid species derived from other brain regions during AD pathogenesis are the direct mediators of the damage to the lateral ventricle/choroid plexus tissue. Indeed the pathobiology of AD starts many years before the manifestation of clinical symptoms, but as we only have access to a snap shot of each patient at autopsy, we are not able to delineate progression of longitudinal changes.

Nonetheless, we can speculate based on our findings and those of others that deficits observed in the lateral ventricles/choroid plexus tissue in AD, are likely to contribute to a deterioration in some of their essential functions (e.g. maintaining BCSFB, CSF production rate, normal CSF pressure and circulation, absorption and drainage) which can impact on brain clearance (of Aβ and tau). In support of this association, studies have implicated a defect in the uptake, transport and processing of AD-related pathogenic proteins by ependymal cells lining the ventricles in AD. For example, alterations in the expression of endocytotic clearance receptor, megalin [58, 64–67], subunit of the ubiquitin proteasome activator complex PA28 [40], and amyloid binding proteins such as transthyretin [58, 68], gelsolin [51], and apolipoprotein J [69], have been described in ependymal cells lining the ventricles in AD. Moreorover, other studies have likewise implicated a defective APP/Aβ processing in ependymal cells in AD [70, 71]. With our studies also supporting proteasomal and lysosomal deficits typified by reductions in Ubiquitin-conjugating proteasome enzyme E2, and a lysosomal ATPase protein subunit.

### Proteomic changes in relationship to amyloid pathology and Braak (tau) staging

Because Alzheimer’s pathology primarily involves tau and amyloid pathogenesis, we further stratified our datasets in all the 26 autopsy cases to demonstrate molecular pathways that particularly correlate with (low vs high) amyloid plaque scoring and (early 0-II vs late IV-VI stage) Braak (phosphorylated [p-tau]) staging. We observed 21 and 24 proteins significantly correlated with amyloid plaque score and Braak (p-tau) staging, respectively. Ingenuity pathway analysis identified oxidative stress, impaired TCA cycle II and related mictochondrial energy metabolism, and impaired amino acid degradation were unique to both tau and amyloid pathologies. Remodeling of the epithelial adherens junctions, Fc *γ* receptor mediated phagocytosis in macrophages and monocytes and RhoA signaling were among the top pathways observed to ‘specifically’ correlate with amyloid score, and could be involved in driving the pathogenesis of amyloidosis or alternatively be a consequence of significant ß-amyloid pathology. Braak staging on the other hand was uniquely correlated with impairments in iron homeostasis signaling pathway, superoxide radical degradation and phagosome maturation, and also implicates these pathological states in either driving tauopathy or as part of the secondary response to hyperphosphorylated tau species in the brain during AD pathogenesis.

### Proteomic changes in nonagenarian patients

Age is the greatest risk factor of AD, thus a novel approach towards investigating the pathogenesis of AD is to interrogate nonagenarians who are resilient to AD pathology and to compare these cases with younger controls and AD cases at autopsy to build a prospective sequelae of events in AD pathobiology. We thus conducted a similar study herein to identify those resilient presumably “successful aging” proteomic profiles in the tissue located around the walls of the inferior horn of the lateral ventricles. Our proteomics analyses identified 22 proteins significantly changing in the control vs nonagenarian group. The Top 3 pathways significantly regulated in this group comparison were changes in phagocytic clearance mechanisms (specifically phagosome maturation), impaired tight-junction signaling involved in mediating proliferation, differentiation, migration, growth/survival pathways, and cytokine signaling, and glucose and mannose metabolism which are key for cellular bioenergetics. These changes could represent a picture of preceding AD pathology and the ‘non-pathological’ stages in the resilient nonagenarian brain, which could be on an early cognitive continuum towards AD. It is noteworthy that most of the nonagenarian cases we investigated exhibited Braak stages of IV-V, despite the low amyloid and CDR score, suggesting that they may be part of the primary age-related tauopathy (PART) spectrum [72–75].

When we interrogated the nonagenarian vs AD group, we observed significant changes in 23 proteins. The top pathways significantly regulated in this group comparison were amyotrophic lateral sclerosis signaling implicating an upregulation in oxidative stress, changes in gluconeogenesis and glycolysis pathways indicating an impairment in mitochondrial bioenergetics, and phosphodiesterase type 5 (PDE5) inhibition which implicates a deficiency in the vasodilatory effect of smooth muscle cells of the highly vascularized stroma that could impact on vascular permeability, inflammation and vascular leakage. These pathobiological changes unique to these group comparisons could represent a novel approach to developing new strategies to mitigate AD-like pathogenesis. However, further studies will be needed in preclinical models to explore their role in AD pathobiology.

### Limitation in attributing protein changes to a distinct cell type

It is worthy of note that the tissue we examined in the inferior horn of the lateral ventricles contained a mixed cell population. We utilized the PanglaoDB database [76] to search for proteins associated with distinct brain cell types. Our assessment of the list of proteins identified in the proteomic experiments indicated numerous proteins associated with epithelial and ependymal cells. However, proteins indicative of other cell types were also identified, such as astrocytes, oligodendrocytes, neurons, endothelia, fibroblasts, dendritic/immune cells. Thus our studies should be interpreted accordingly, as the significant changes in proteins identified may originate from different cell types and not only the ependymal/epithelial cells.

Intriguingly, we were able to demonstrate that of the 7 different brain cell types, most of the significant changes in cell-specific proteins observed between controls vs nonagenarian cases were associated mainly with ependymal cells. While in nonagenarians vs AD cases ependymal cells and neurons ranked highest. Future work using single cell isolation from fresh tissue would be interesting to specifically identify single cell changes at the protein (LC-MS/MS or IHC) and/or gene (e.g. 10x genomics or microdissection) levels in a similar cohort.

## Conclusion

In this study, we hypothesized that molecular aberrations (at the protein level) in the unique cell types lining the walls of the lateral ventricles and nearby surrounding regions, may compromise their physiological properties and essential functions, which could act as a medium for contributing towards AD pathogenesis. We identified top molecular pathways significantly altered in AD pathogenesis involving impaired mitochondrial bioenergetics, oxidative stress, remodeling of epithelia adherens junction barrier, recruitment of macrophage/monocytes and increase in their phagocytic activity, and alterations in cytoskeletal dynamics. These appeared to be driven by upstream regulators PPARGC1A, 14–3-3 protein *γ*, PSEN1, MAPT and APP genes. Oxidative stress, impaired amino-acid metabolism, and mitochondrial bioenergetics were observed in the AD tissue irrespective of tau or amyloid staging. Additionally, remodeling of the epithelial adherens tight junctions, Fc *γ* receptor mediated phagocytosis in macrophages and monocytes, and RhoA signaling and its impact of cytoskeletal dynamics were unique and specific to amyloid pathology. While Tau pathology was uniquely correlated with altered iron homeostasis signaling pathway, superoxide radical degradation and phagosome maturation.

Although our studies are unable to determine whether disrupted cellular functions in the lateral ventricles precede AD pathology, it appears that significant molecular changes within this brain region may be, at least, one of the contributing factor in AD pathogenesis. As there are multifactorial changes observed in AD, which can also be compounded by genetic (e.g. apolipoprotein E4) and environmental insults (e.g. brain injury), more studies in a larger prospective cohort are need to elucidate the role of this brain region in AD. In particular, developing in vivo imaging methods to assess brain ventricle/choroid plexus function and CSF biofluid dynamics in living patients at pre, peri and post onset of pathology. Our findings are also significant because considering that some of the proteins identified can be secreted or released into the extracellular environment, they could therefore serve as potential biomarkers in the blood/CSF for AD pathogenesis, particularly those changing at the earliest and most crucial stages or inflection point of the disease (i.e. those unique to nonagenarians or AD).

## Supplementary information

**Additional file 1: Table S1.** Disease and Biofunctions modulated in the inferior horn of the lateral ventricles of AD patients, their aged-matched controls and nonagenarians. Underlying disease pathology and biofunctions generated from the list of significantly modulated proteins across three groups of interest using Ingenuity pathway analyses (IPA). Value represents FDR adjusted *p* value after Fischer’s test and Benjamin Hochberg correction following uploading of Log2Ratio values of significantly regulated proteins into IPA. Red box – downregulated across group; Blue box – upregulated across group.

**Additional file 2: Table S2.** Disease and Biofunctions modulated in the inferior horn of the lateral ventricles after stratification for Braak staging and amyloid plaque score. Underlying disease pathology and biofunctions generated from the list of significantly modulated proteins after stratification for Braak staging (0-II vs IV-VI) and amyloid plaque score using Ingenuity pathway analyses (IPA). Value represents FDR adjusted *p* value after Fischer’s test and Benjamin Hochberg correction following uploading of Log2Ratio values of significantly regulated proteins into IPA. Red box – downregulated across group; Blue box – upregulated across group.

**Additional file 3: Figure S1.** Validation of NEFM, NFH and SOD1 by western blotting. Validation of proteomic changes using antibody based methods. Figure shows comparisons of changes in expression of three proteins (Neurofilament medium chain polypeptide [NEFM] – AA’, Neurofilament medium chain polypeptide [NF-H] – BB’, Superoxide dismutase – CC’) in AD, age-matched controls and nonagenarian cases between antibody based measurement using western blotting and our proteomic LC-MS/MS analyses. Data(±SEM) represents intensity values normalized to total protein from stain-free gels. Immunoblotting images (DD’) and stain free gel images (EE’) are depicted for membrane/gel 1# and membrane 2# (respectively). Sample size *N* = 6 control (C); *N* = 9 nonagenerian (N) and *N* = 11 AD (A). The first 3 lanes in gel E/E’ contained the same reference samples for normalization, these lanes are cropped in D/D’. Samples with Low total abundant proteins in the gel were not analyzed.

## Data Availability

The datasets used and/or analyzed during the current study available from the corresponding author on reasonable request.

## Abbreviation

ATP5L: Adenosine triphosphate
ALDH: Aldehyde dehydrogenase
AD: Alzheimer’s disease
APBA3: Amyloid precursor protein
APP: Amyloid precursor protein
Aß: Amyloid-ß protein
ANOVA: Analysis of variance
APOE4: Apolipoprotein E4
BCSFB: Blood-CSF barrier
CSF: Cerebrospinal fluid
CDR: Clinical dementia rating
CERAD: Consortium to Establish a Registry for Alzheimer’s Disease
DDA: Data dependent acquisition
E1F2: Eukaryotic Initiation Factor 2
FDR: False discovery rate
GEO: Gene Expression Omnibus
GFAP: Glial fibrillary acidic protein
Hsp: Heat shock protein
HO1: Heme oxygenase-1
IHC: Immunohistochemistry
IPA: Ingenuity pathway analyses
IRB: Institutional Review Board
IFN-I: Interferon type I
IL1RL1: Interleukin 1 receptor like 1
IL1R: Interleukin-1 receptor
ISF: Interstitial fluid
JAK-STAT: Janus Kinase and Signal transducer and activator of transcription protein
mTOR: Mammalian target of rapamycin
LC-MS: Liquid chromatography/mass spectrometry
NFT: Neurofibrillary Tangle
NEFM: Neurofilament medium chain polypeptide
NF-H: Neurofilament medium chain polypeptide
NO: Nitric oxide
NRF2: Nuclear factor erythroid 2-related factor 2
PBS: Phosphate buffer saline
PDE5: Phosphodiesterase type 5
p-tau: Phosphorylated tau protein
PART: Primary age-related tauopathy
PA28: proteasome activator complex
S100 *β*: S100 calcium-binding protein B
NaCl: Sodium Chloride
SDC: Sodium deoxycholate
SLC4A5: Solute carrier gene
TMT: Tandem mass tags
TCA: Tricarboxylic acid cycle
TEAB: Triethylamonium bicarbonate
UPLC: Ultra-Performance Liquid Chromatography
