# Supplementary figures and images for: Molecular abnormalities in autopsied brain tissue from the inferior horn of the lateral ventricles of nonagenarians and Alzheimer disease patients
Source: BMC Neurol. 2020 Aug 27;20:317. doi: 10.1186/s12883-020-01849-3 (PMC7450601; doi:10.1186/s12883-020-01849-3)

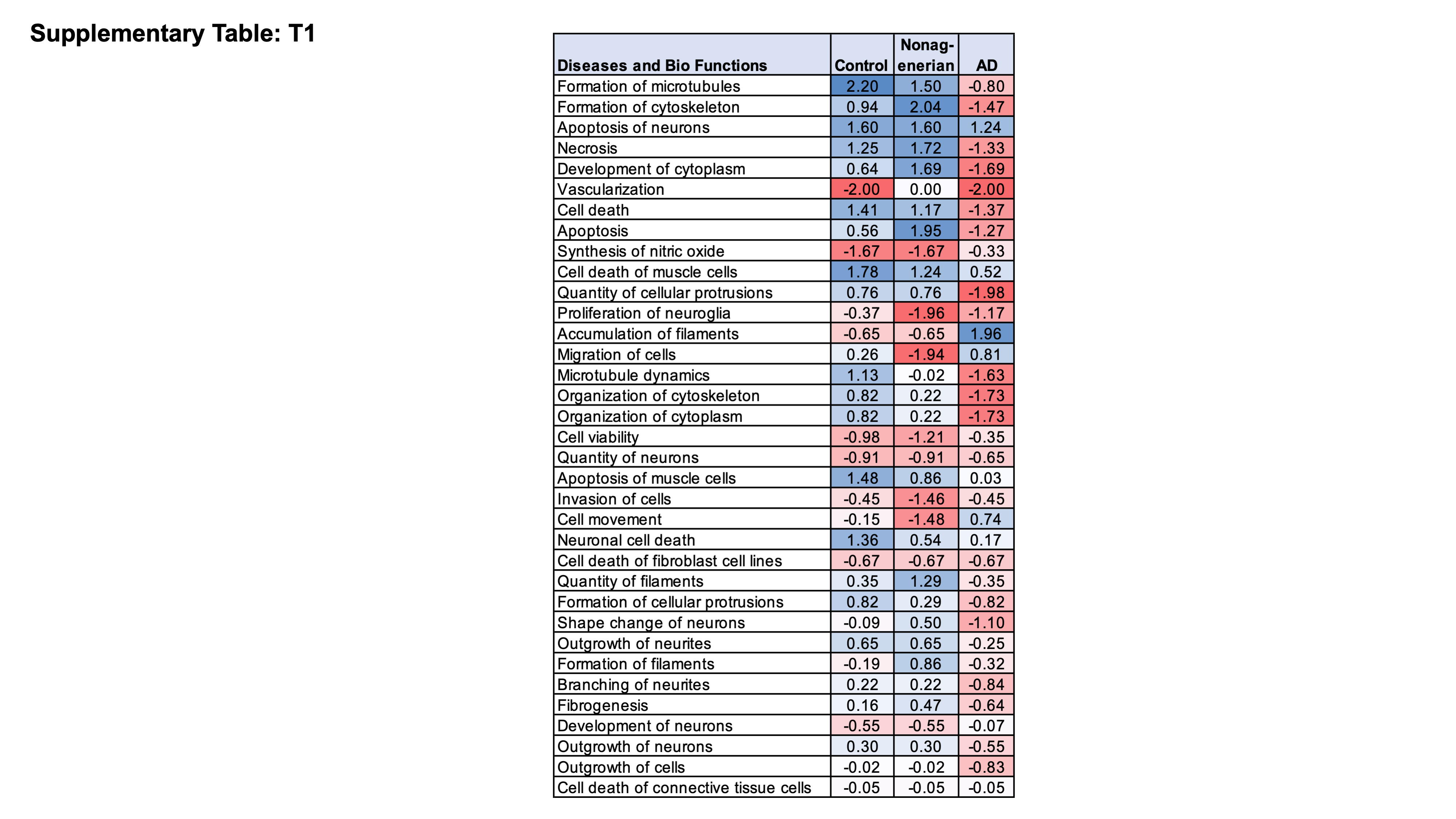

Supplement: Supplementary file 1 — Additional file 1: Table S1. Disease and Biofunctions modulated in the inferior horn of the lateral ventricles of AD patients, their aged-matched controls and nonagenarians. Underlying disease pathology and biofunctions generated from the list of significantly modulated proteins across three groups of interest using Ingenuity pathway analyses (IPA). Value represents FDR adjusted p value after Fischer’s test and Benjamin Hochberg correction following uploading of Log2Ratio values of significantly regulated proteins into IPA. Red box – downregulated across group; Blue box – upregulated across group. [file 12883_2020_1849_MOESM1_ESM.tiff]

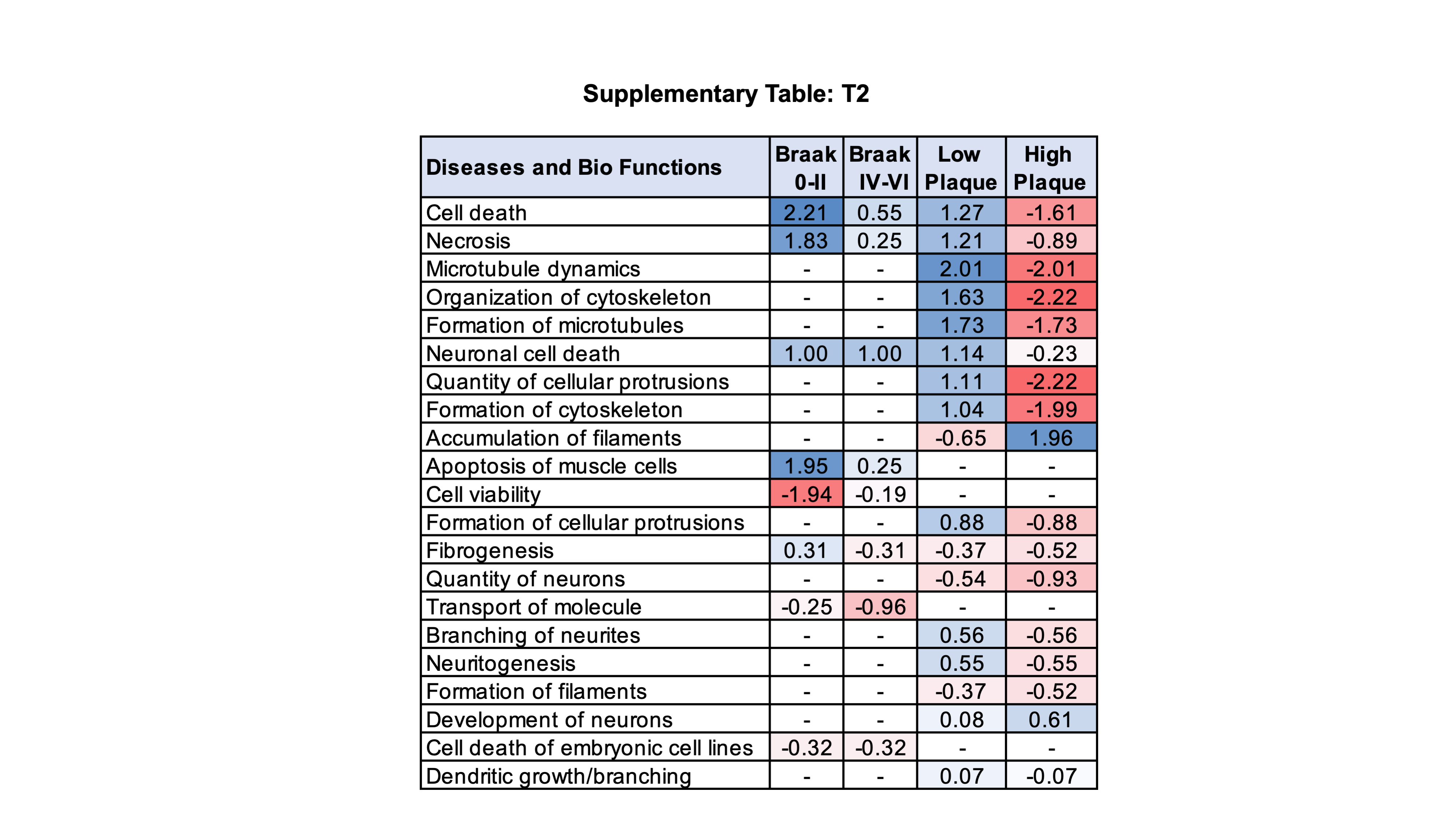

Supplement: Supplementary file 2 — Additional file 2: Table S2. Disease and Biofunctions modulated in the inferior horn of the lateral ventricles after stratification for Braak staging and amyloid plaque score. Underlying disease pathology and biofunctions generated from the list of significantly modulated proteins after stratification for Braak staging (0-II vs IV-VI) and amyloid plaque score using Ingenuity pathway analyses (IPA). Value represents FDR adjusted p value after Fischer’s test and Benjamin Hochberg correction following uploading of Log2Ratio values of significantly regulated proteins into IPA. Red box – downregulated across group; Blue box – upregulated across group. [file 12883_2020_1849_MOESM2_ESM.tiff]

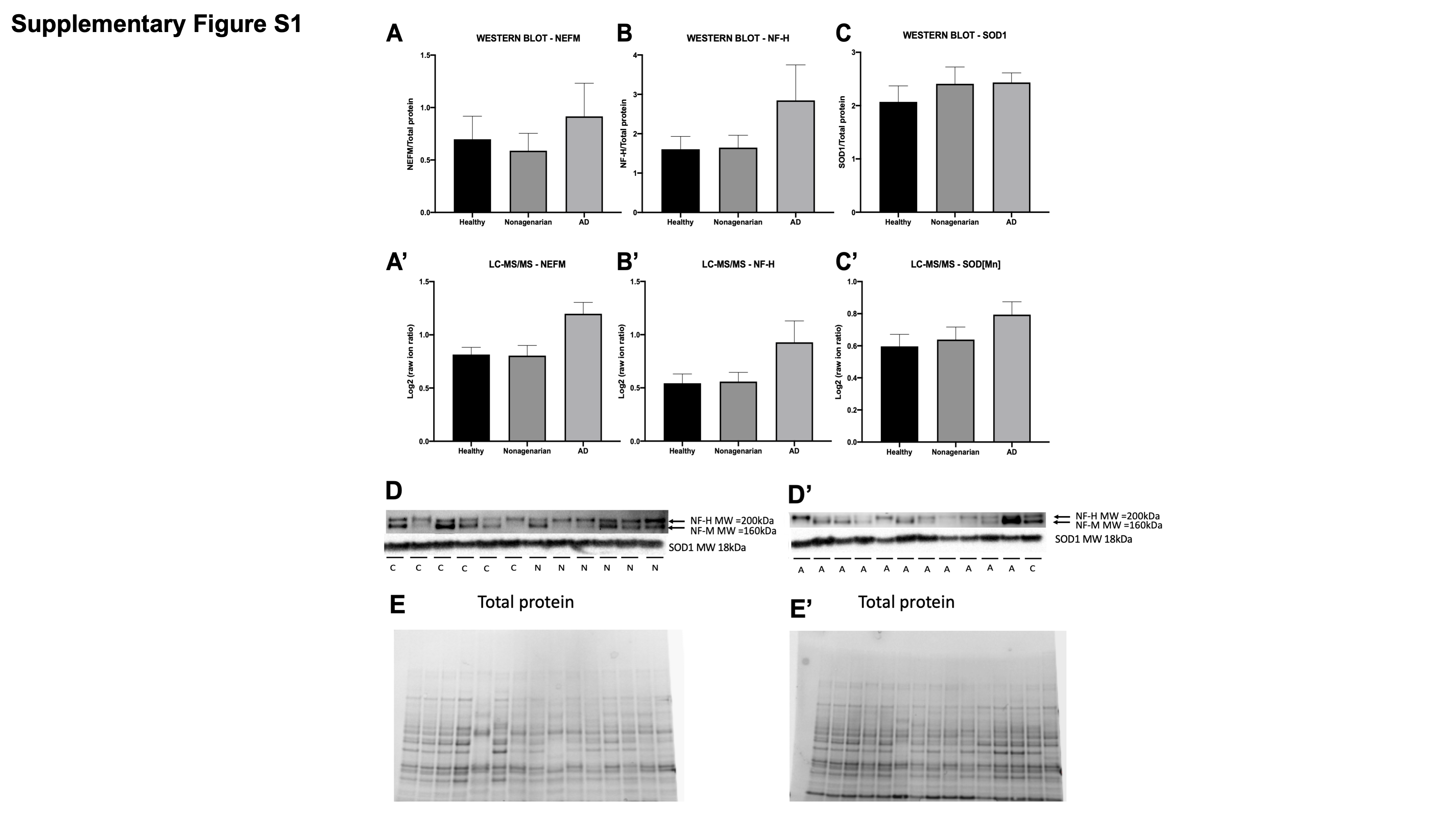

Supplement: Supplementary file 3 — Additional file 3: Figure S1. Validation of NEFM, NFH and SOD1 by western blotting. Validation of proteomic changes using antibody based methods. Figure shows comparisons of changes in expression of three proteins (Neurofilament medium chain polypeptide [NEFM] – AA’, Neurofilament medium chain polypeptide [NF-H] – BB’, Superoxide dismutase – CC’) in AD, age-matched controls and nonagenarian cases between antibody based measurement using western blotting and our proteomic LC-MS/MS analyses. Data(±SEM) represents intensity values normalized to total protein from stain-free gels. Immunoblotting images (DD’) and stain free gel images (EE’) are depicted for membrane/gel 1# and membrane 2# (respectively). Sample size N = 6 control (C); N = 9 nonagenerian (N) and N = 11 AD (A). The first 3 lanes in gel E/E’ contained the same reference samples for normalization, these lanes are cropped in D/D’. Samples with Low total abundant proteins in the gel were not analyzed. [file 12883_2020_1849_MOESM3_ESM.tiff]
